# Total alveolar lavage with oxygen fine bubble dispersion directly improves lipopolysaccharide-induced acute respiratory distress syndrome of rats

**DOI:** 10.1038/s41598-020-73768-9

**Published:** 2020-10-06

**Authors:** Kenta Kakiuchi, Takehiro Miyasaka, Shinji Takeoka, Kenichi Matsuda, Norikazu Harii

**Affiliations:** 1 Department of Life Science and Medical Bioscience, Graduate School of Advanced Science and Engineering, Waseda University (TWIns), Tokyo, 162-8480 Japan; 2 Department of Materials and Human Environmental Science, Shonan Institute of Technology, Kanagawa, 251-0046 Japan; 3 Department of Emergency and Critical Care Medicine, University of Yamanashi School of Medicine, 1110 Shimokato, Chuo-shi, Yamanashi 409-3898 Japan

**Keywords:** Diseases, Respiratory tract diseases, Health care, Therapeutics, Medical research, Experimental models of disease

## Abstract

Severe respiratory disorder induced by pulmonary inflammation is one of the causes of acute respiratory distress syndrome, which still has high mortality. It is crucial to remove causative substances and inflammatory mediators early in order to inhibit the progression of pulmonary inflammation. Total alveolar lavage (TAL) may avert the inflammatory response by eliminating causative substances in certain inflammatory lung diseases. We developed an efficient TAL system and examined the efficacy of short-term TAL treatment performed for acute lung injury models of rats. In the first experiment with a severe lung injury model, 15 rats were divided into 3 groups: sham group, mechanical gas ventilation (MGV) treatment group, and TAL treatment group. The treatments were conducted for 5 min, 20 min after the provocation of inflammation. Two days after treatment, the TAL and MGV treatment groups exhibited significant differences in blood oxygen levels, mean arterial pressure, weight-loss ratio, and inflammatory cytokine levels in the lungs. In contrast, almost no differences were observed between the TAL treatment and sham groups. In the second experiment with a lethal lung injury model, the TAL treatment dramatically improved the survival rate of the rats compared to the MGV treatment groups (*p* = 0.0079). Histopathological analysis confirmed pronounced differences in neutrophil accumulation and thickening of the interstitial membrane between the TAL and MGV treatment groups in both experiments. These results indicate that as little as 5 min of TAL treatment can protect rats from acute lung injury by removing causative substances from the lungs.

## Introduction

Acute respiratory distress syndrome (ARDS) is a rapidly progressive disease, and consists of two categories grouped by underlying disease: direct ARDS following pulmonary pathology and indirect ARDS following systemic inflammation^1^. The characteristics of ARDS are diffuse alveolar damage, intra-alveolar infiltration of inflammatory cells, oversecretion of cytokines, and thickening of the interstitial membrane^2^. Since these pathological alterations may result in irreversible respiratory failure, it is crucial to remove the causative substances and protect patients from the inflammatory progression at an early phase. Particularly, in direct ARDS, the progression of systemic inflammation should be avoided. However, no treatment to directly improve the severe lung inflammation has been established and supportive respiratory management remains the primary treatment^3^.

Lung lavage with saline was proposed by Winternitz and Smith in 1918. They showed that pre-installed India ink, starch paste, and non-pathogenic bacteria could be removed by lung lavage in dogs, with bacterial load reduced by 90%^4^. After that, the therapeutic effects of lung lavage were reported, mainly as case reports, for some lung diseases such as: pulmonary alveolar proteinosis, asthma, cystic fibrosis, bronchiectasis, and lipoid pneumonia^5–8^. However, some patients displayed ventilatory impairment and fever; furthermore, respiratory management after the treatment was challenging. These reports summarised that lung lavage was effective for the symptoms induced by secretion accumulation or alveolar blockage, but not for those caused by structural failure^5,6,9^.

In our previous study, we constructed a total liquid ventilation (TLV) system with an oxygen fine bubble dispersed saline (FB dispersion)^10^. TLV is one of the artificial respiration mechanisms using liquid, where gas exchange is facilitated by continuous liquid replacement^11^. FB dispersions are a simple, inexpensive oxygen carrier and able to contain more oxygen than oxygen-saturated water. We reported that anaesthetised rats survived over 40 min with the FB dispersion, versus 7.5 min with air-saturated saline and 22.6 min with oxygen-saturated saline^10^. Although these results showed the FB dispersion was a hopeful material for TLV, the oxygen content used was insufficient to perform ordinal gas exchange during TLV. However, TLV systems can re-expand the collapsed alveoli with low pressure and wash the whole alveoli in a short time^12,13^. Therefore, we developed the total alveolar lavage (TAL) system based on the TLV system which can conduct extensive alveolar lavage within a short time.

In this study, we aimed to verify the efficacy of the TAL with the new system (TAL treatment) in acute lung injury rat models induced by intratracheal lipopolysaccharide (LPS) administration. An experimental protocol was constructed with the hypothesis that a 5-min TAL session is sufficient to be therapeutic by removing LPS and reducing their harmful effects on the lungs.

## Results

### Experiment 1: the efficacy of short-term total alveolar lavage in a severe respiratory failure model

**Figure 1:**
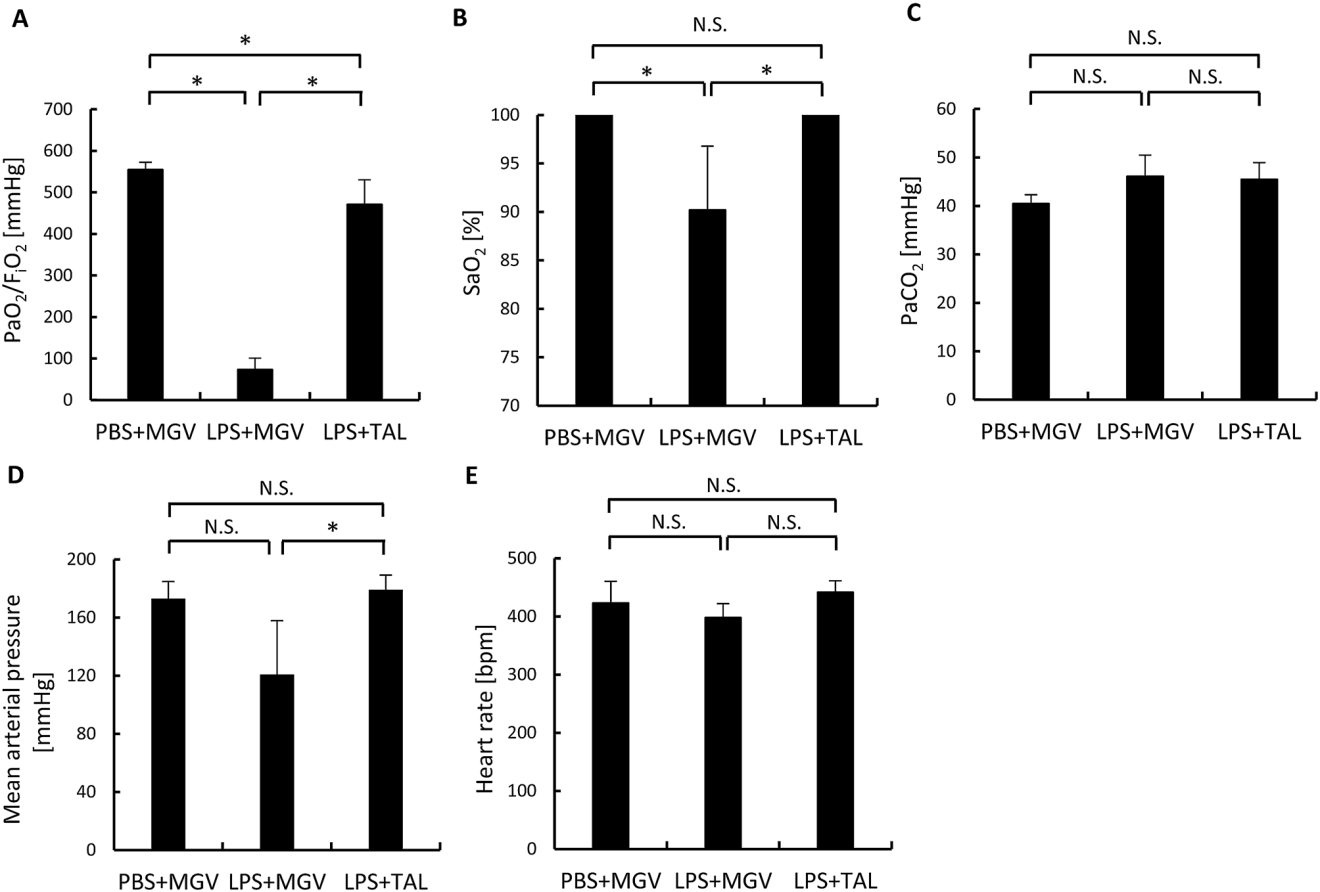
Physiological conditions of the rats 2 days after each treatment. (**A**) Arterial oxygen partial pressure /fractional inspired oxygen concentration (PaO_2_/FIO_2_), (**B**) arterial oxygen saturation (SaO_2_), and (**C**) arterial carbon dioxide partial pressure (PaCO_2_) were measured in blood gas analyses at 2 days after each treatment in the PBS + MGV, LPS + MGV, and LPS + TAL groups; n = 5 rats/group. (**D**) The mean arterial blood pressure (10 min average) and (**E**) heart rate (10 min average) were measured 2 days after each treatment in the PBS + MGV, LPS + MGV, and LPS + TAL groups; n = 5 rats/group. Data are presented as the mean ± standard deviation; *p < 0.05. N.S. indicates not significant.

Figure 1 shows the physiological conditions 2 days after each treatment in the 3 groups: phosphate-buffered saline (PBS) + MGV group (sham group), LPS + MGV group (MGV treatment group), and LPS + TAL group (TAL treatment group). Arterial oxygen partial pressure/fractional inspired oxygen concentration (PaO_2_/FIO_2_) in the LPS + TAL group was significantly higher than that in the LPS + MGV group and significantly lower than that in the PBS + MGV group (Fig. 1A, *p* < 0.05). Arterial oxygen saturation (SaO_2_) of all rats in the LPS + TAL and PBS + MGV groups was 100% and significantly higher than that in the LPS + MGV group, which was 90% (Fig. 1B, *p* < 0.05). Blood oxygenation levels in the LPS + TAL and PBS + MGV groups were within the normal range whereas that in the LPS + MGV group indicated severe hypoxemia. There were no significant differences in arterial carbon dioxide partial pressure (PaCO_2_) among all groups, although a few rats in the LPS + MGV and LPS + TAL groups showed a slightly acidic blood condition (Fig. 1C). Mean arterial pressure (MAP) in the LPS + TAL group was significantly higher than that in the LPS + MGV group (Fig. 1D, *p* < 0.05), and there were no significant differences between the LPS + TAL and PBS + MGV groups (Fig. 1D). There were no significant differences in heart rate among all groups (Fig. 1E). Haemodynamic disorders were confirmed only in the LPS + MGV group.

**Figure 2:**
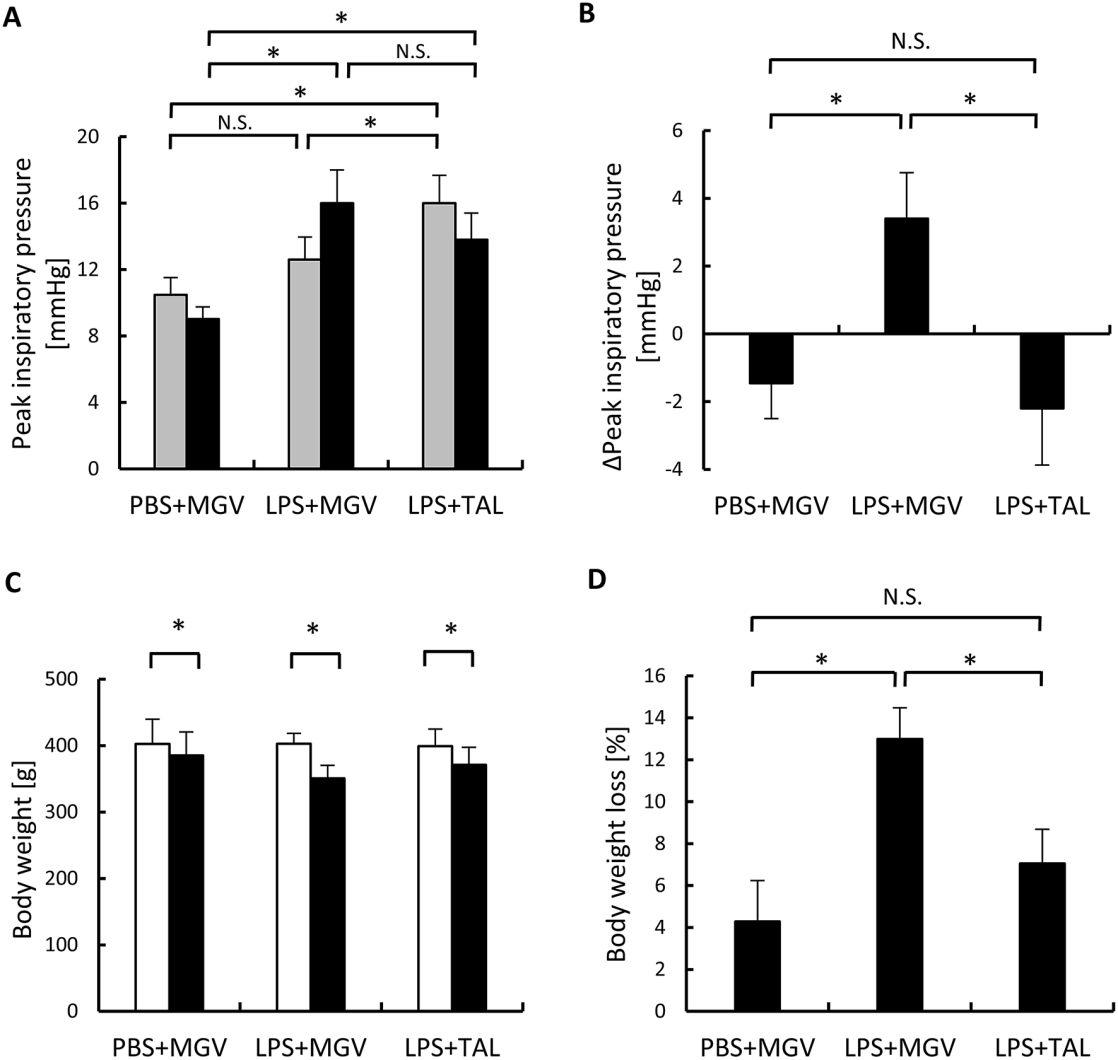
The changes in peak inspiratory pressure and body weight between day 0 and day 2. (**A**) The peak inspiratory pressure at day 0 (just after treatment) and 2 days after each treatment in the PBS + MGV, LPS + MGV, and LPS + TAL groups are shown; n = 5 rats/group (grey bar: day 0 (after treatment), black bar: day 2). (**B**) The degree of changes in the peak inspiratory pressure (∆Peak inspiratory pressure [PIP]) in the PBS + MGV, LPS + MGV, and LPS + TAL groups; n = 5 rats/group. ∆Peak inspiratory pressure = PIP at day 0 – PIP at day 2. (**C**) The bodyweight of the rats at day 0 (just before treatment) and 2 days after each treatment in the PBS + MGV, LPS + MGV, and LPS + TAL groups; n = 5 rats/group (white bar: day 0 (before treatment), black bar: day 2). (**D**) The percentage of body weight loss in the PBS + MGV, LPS + MGV, and LPS + TAL groups; n = 5 rats/group. Data are presented as the mean ± standard deviation; *p < 0.05.

Parameter changes between day 0 and day 2 in the 3 groups are shown in Fig. 2. Peak inspiratory pressure (PIP) at day 0 (after each treatment) in the LPS + TAL group was significantly higher than those in the other 2 groups, which were not significantly different from each other (Fig. 2A grey bar, *p* < 0.05). PIP at day 2 in the PBS + MGV group was significantly lower than those in the other 2 groups (Fig. 2A black bar, *p* < 0.05). The degree of changes in the PIP between day 0 and day 2 (∆PIP) showed a positive value in the LPS + MGV group and negative values in both LPS + TAL and PBS + MGV groups (Fig. 2B). There were significant differences between the ∆PIP in the LPS + MGV group and the other 2 groups (Fig. 2B, *p* < 0.05). Only in the LPS + MGV group, the condition inside the lungs deteriorated within 2 days, and the airway pressure increased. The body weights of all rats significantly decreased 2 days after each treatment (Fig. 2C, *p* < 0.05). However, the percentage of weight loss in the LPS + MGV group was significantly larger than those in the other 2 groups (Fig. 2D, *p* < 0.05).

**Figure 3:**
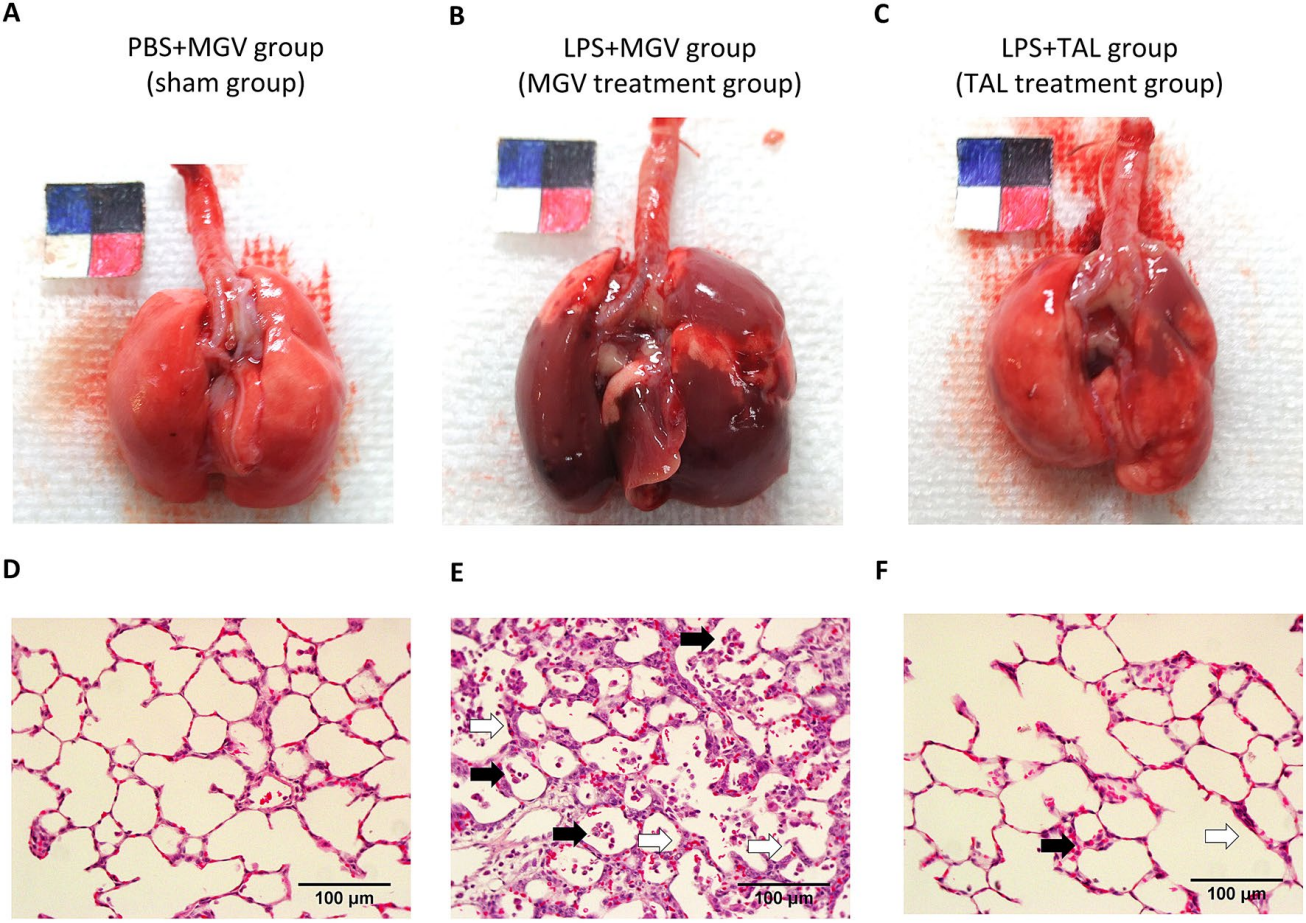
The macro- and histopathological findings of the lungs 2 days after each treatment. The representative pictures of the lungs in the (**A**) PBS + MGV (**B**) LPS + MGV, and (**C**) LPS + TAL groups. The left top sheet is a combination of colorimetric paper and length scale with a 10-mm^2^ square (5-mm^2^ squares in 4 colours). The representative microscopic images of the lung tissue section (posterior lobe) stained with hematoxylin and eosin (H&E) in the (**D**) PBS + MGV (**E**) LPS + MGV, and (**F**) LPS + TAL groups. Scale bar, 100 µm (Magnification, ×400). Black arrows, neutrophil accumulation and white arrows, thickened alveolar walls.

Figure 3 shows the macroscopic findings of the lungs and the histopathological findings for each lung tissue. Homogenous severe inflammation was confirmed in the LPS + MGV group (Fig. 3B). However, mild inflammation was observed in the LPS + TAL group, and no inflammatory features were observed in the PBS + MGV group (Fig. 3A,C). At the tissue level, the LPS + MGV group showed the typical signs of inflammation: accumulation of neutrophils in interalveolar spaces, thickened alveolar walls (Fig. 3E). These findings were noticeably suppressed in the LPS + TAL group, which showed only mild inflammatory features (Fig. 3F). The PBS + MGV group showed normal findings (Fig. 3D).

**Figure 4:**
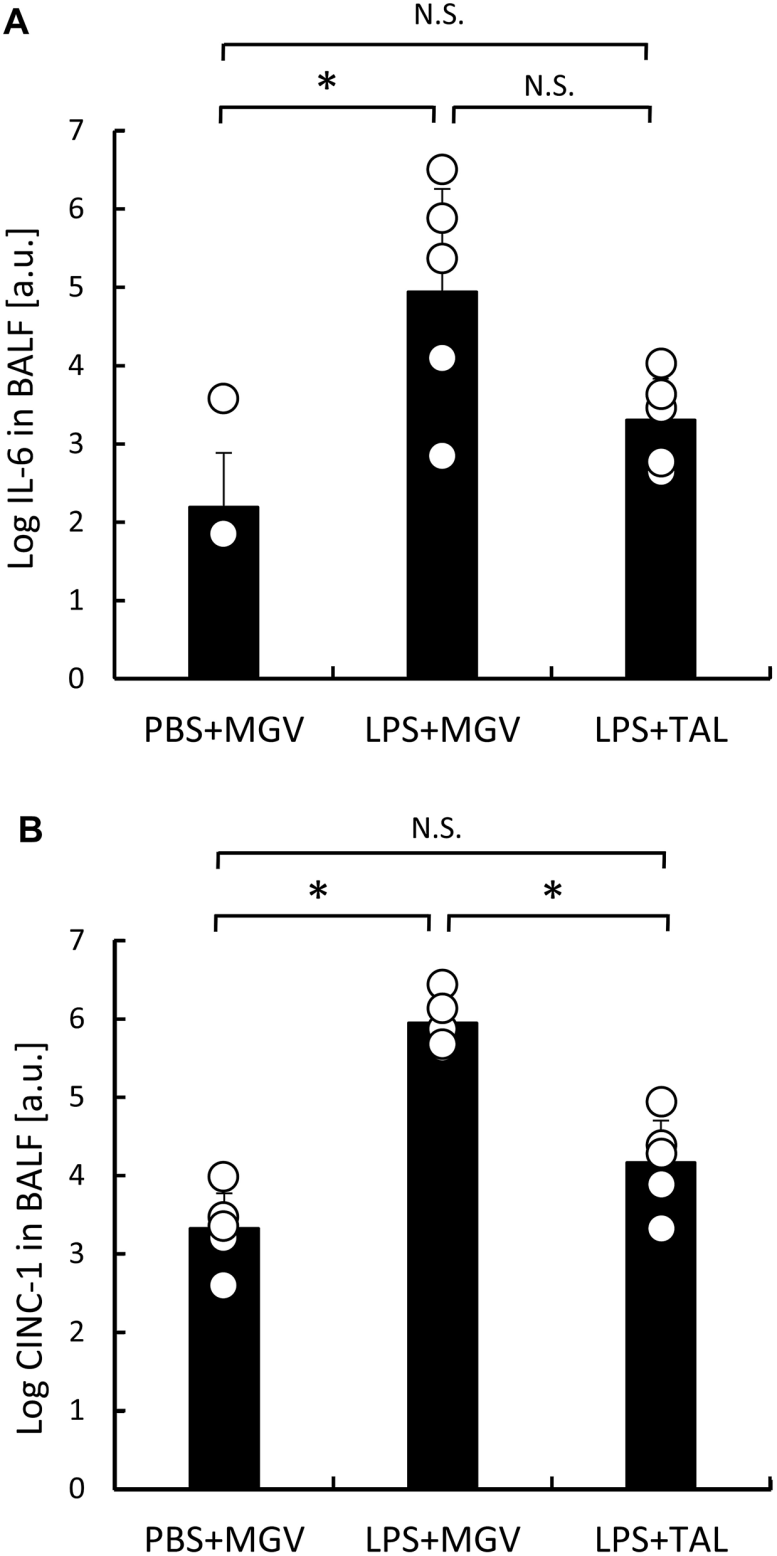
The concentrations of inflammatory cytokines and chemokines two days after each treatment. The concentrations of (**A**) IL-6 and (**B**) CINC-1 in the BALF in the PBS + MGV, LPS + MGV, and LPS + TAL groups; n = 5 rats/group. The results are shown as natural logarithmic values. The hollow circle represents each measurement value in a group. Data are presented as mean ± standard deviation; *p < 0.05.

Inflammatory cytokine levels in the bronchoalveolar lavage (BAL)-fluid (BALF) were measured with an enzyme-linked immunosorbent assay (ELISA) and expressed as natural logarithmic values in Fig. 4. The interleukin-6 (IL-6) level in the LPS + MGV group was significantly higher than that in the PBS + MGV group (Fig. 4A, *p* < 0.05), and the IL-6 level in the LPS + TAL group tended to be located in between those in the PBS + MGV and LPS + MGV groups (Fig. 4A). The concentration of cytokine-induced neutrophil chemoattractant 1 (CINC-1), which induces accumulation of neutrophils, in the LPS + MGV group was significantly higher than in the other 2 groups, which showed no significant difference (Fig. 4B, *p* < 0.05). Inflammatory cytokine levels were increased in the LPS + MGV group and suppressed in the LPS + TAL group. These results showed the same tendency as the macro- and microscopic findings in Fig. 3.

### Experiment 2: the efficacy of short-term total alveolar lavage in a lethal respiratory failure model

**Figure 5:**
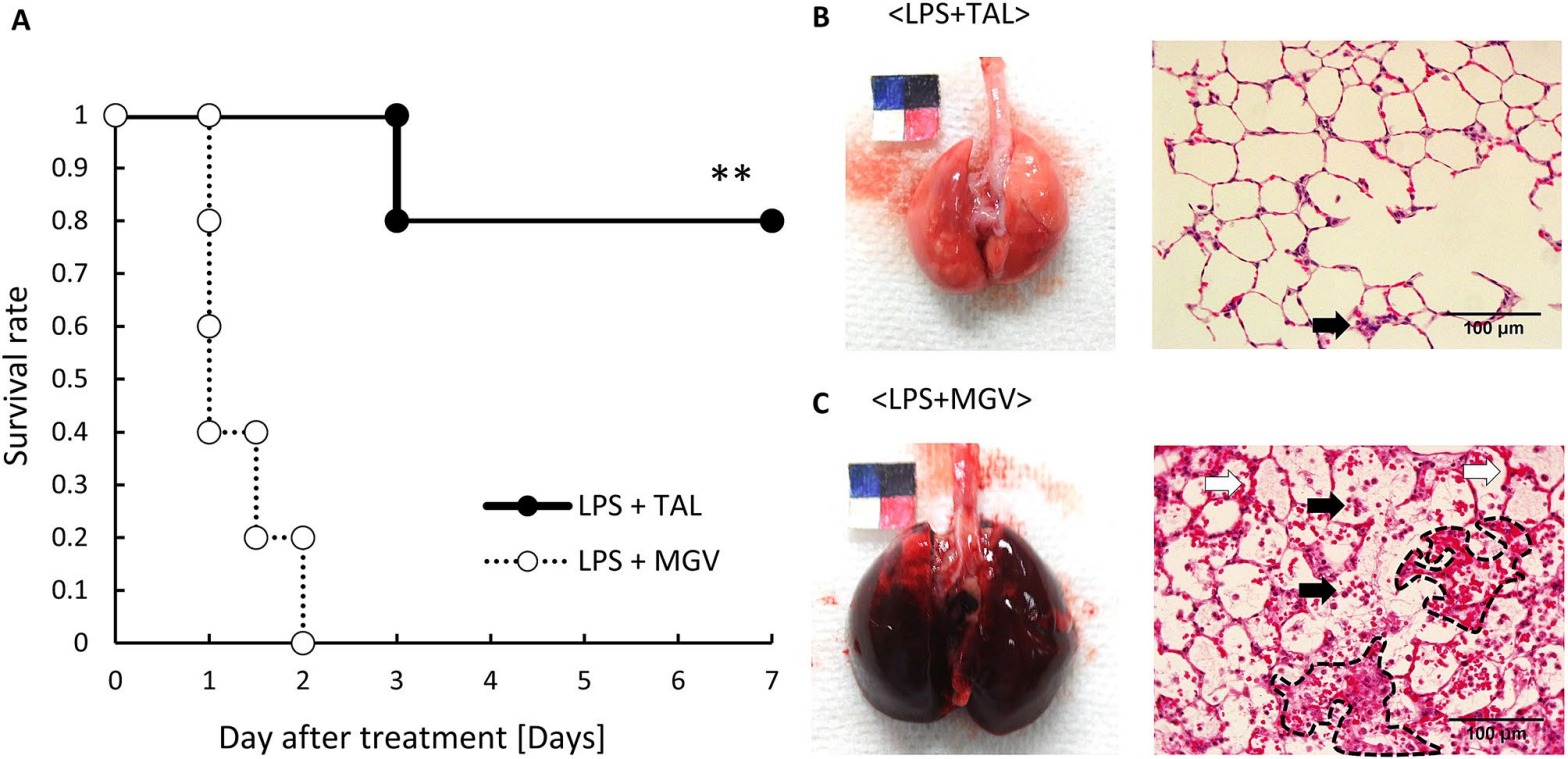
The survival rate and the macro- and histopathological findings of the lungs after each treatment. (**A**) The Kaplan–Meier curve of the rats in the LPS + MGV and LPS + TAL groups (hollow circle, LPS + MGV group; closed circle, LPS + TAL group; n = 5 rats/group, **p < 0.01). The representative pictures of the lungs and histopathological findings of the lung tissue (posterior lobe) in the (**B**) LPS + TAL group and (**C**) LPS + MGV group. The left top sheet is a combination of colorimetric paper and length scale with a 10-mm^2^ square (5-mm^2^ squares in 4 colours). Scale bar, 100 µm. (Magnification, ×400) Black arrows, neutrophil accumulation; white arrows, thickened alveolar walls; dotted areas, haemorrhage.

All rats in the LPS + MGV group died within 2 days after the administration of 10 mg/kg of LPS. Contrastingly, the survival rate of the rats significantly improved to 80% with the TAL treatment (LPS + TAL group) (Fig. 5A, *p* = 0.0079). The lungs of the dead rats in the LPS + MGV group 2 days after LPS administration showed homogenous severe congestion and hypertrophy (Fig. 5C, left panel). The histopathological findings in the LPS + MGV group showed an accumulation of neutrophils along with severe alveolar haemorrhage (Fig. 5C, right panel). Both macro- and microscopic findings in the LPS + TAL group indicated almost healthy and well-preserved alveolar structures (Fig. 5B). In addition, haemodynamic (MAP: 173 ± 15.6 mmHg, HR: 431 ± 46.6 bpm) and blood oxygenation (PaO_2_/F_i_O_2_: 505 ± 50.9, SaO_2_: 100%) data of the surviving rats in the LPS + TAL group were within the normal range at 1 week after the TAL treatment (Table 1).

**Table 1:** Physiological conditions of the rats 7 days after TAL treatment in experiment 2.

groups	MAP (mmHg)	HR (BPM)	pH (−)	PaO_2_ (torr)	PaCO_2_ (torr)	SaO_2_ (%)	PIP (mmHg)
LPS + TAL	173 ± 15.6	431 ± 46.6	7.44 ± 0.05	506 ± 44.1	47.4 ± 2.02	100 ± 0	10.5 ± 1.73
LPS + MGV	N/A	N/A	N/A	N/A	N/A	N/A	N/A

Data are presented as the mean ± standard deviation (n = 4: one rat died from n = 5). N/A indicates not applicable because all rats died within 2 days. MAP: mean arterial pressure; HR: heart rate; PaO_2_: arterial partial oxygen pressure; PaCO_2_: arterial partial carbon dioxide pressure; SaO_2_: arterial oxygen saturation; PIP: peak inspiratory pressure, LPS: lipopolysaccharide, TAL: total alveolar lavage, MGV: mechanical gas ventilation, BPM: beats per minute, pH: potential of hydrogen.

## Discussion

In the present study, we evaluated the efficacy of a 5-min TAL treatment for acute lung injury models. The results demonstrate that severe inflammation was avoided with the TAL treatment, which removed LPS from the lungs. We use LPS to induce direct ARDS because the effect of intratracheal administration of LPS (endotoxin from gram-negative bacteria)^14,15^, on the lungs is well established as a model of ARDS in several animal species^16^. In particular, LPS administration in animal models has been shown to closely mimic the condition of human ARDS^15,17^. In the experiments presented here, the therapeutic effect was attributable to the direct elimination of LPS; however, we postulate that the TAL treatment has the potential to remove other pathogens (bacteria and virus) and inflammatory mediators (cytokines and chemokines) because the findings from BAL studies show that they can be washed out from the lungs with saline^18–20^.

Whole lung lavage (WLL) is the current lung lavage technique of choice for pulmonary alveolar proteinosis used in the clinical field. While this approach is difficult to apply in cases with severe respiratory failure due to risk of hypoxia, studies have shown that 10–15 washes should be enough to remove the secretions from the lungs^21–23^. WLL is performed by using a double-lumen endobronchial tube and washing one lung at a time. It takes 2–6 h for one lung and, in general, a 1–2 week interval is necessary between each lung^24–26^. Since the TAL system can perform TAL in 5 min and treat both lungs simultaneously, we attempted a total of 33 washes (> 15 washes) in this experiment. It is calculated that 10 min would be a sufficient TAL treatment time for human patients.

In experiment 1, at 2 days after LPS administration, the rats in the MGV treatment group showed severe respiratory failure that resulted in severe hypoxia, which is a typical symptom of ARDS^27^. The decreases in blood pressure and body weight indicated dehydration and decreased food intake. Inflammatory obstruction of the alveoli caused an increase in the airway pressure after 2 days, and, the levels of inflammatory cytokines (IL-6 and CINC-1) in the BALF and the macro- and microscopic findings indicated that inflammation had qualitatively and quantitatively progressed in the MGV treatment group. IL-6 is one of the most active pro-inflammatory cytokines and shows elevated levels in association with pathological disorders such as ARDS^28,29^. CINCs, especially CINC-1 and CINC-3, are chemokines that contribute to the neutrophil accumulation and are known to increase during LPS-induced pulmonary inflammation^17,29^.

On the other hand, the TAL treatment group showed an improvement in the hypoxic conditions and suppressed inflammatory responses in comparison with the MGV treatment group. Some parameters in the TAL treatment group were similar to those in the sham group, which did not show inflammation. The PIP in the TAL treatment group immediately after the TAL treatment showed the highest value among the 3 groups, which could be due to the remnant FB dispersion that obstructed some alveoli. However, this is not a severe side effect since a value of 16 mmHg (21.8 cmH_2_O) is not considered to be high enough to damage alveolar structures^30^ and the PIP improved with capillary absorption and vaporisation of the remnant FB dispersion. Consequently, all rats in the LPS + TAL group recovered spontaneous breathing. While the efficacy of the TAL treatment was confirmed from these results, other experiments revealed that the rats in the MGV treatment group recovered at day 7 (data not shown), even though they showed severe respiratory failure on day 2. Therefore, we conducted the experiment 2 with a more severe condition.

In experiment 2, the survival rate in the TAL treatment group was substantially higher than that in the MGV treatment group. There were also distinct differences between the two groups in the histopathological analyses, especially with respect to haemorrhage. Therefore, the TAL treatment successfully suppressed inflammatory deterioration and showed significant efficacy in preventing respiratory failure.

It is also interesting to note that the rats recovered spontaneous breathing following TAL treatment without a combination of lung surfactants or alternative drugs. In general, lung lavage with normal saline is used to prepare a lung surfactant-deficient model^31–33^. We expected that lung surfactants would be required before the rats returned to spontaneous breathing. However, the rats recovered to MGV from the TAL, and ventilation weaning was successful after respiratory management for 3 h. Sufficient drainage of the FB dispersion from the lungs after TAL is an essential aspect of this returning operation. If the drainage were insufficient, the rats would die within 3 h. In this experiment, water absorption after TAL treatment was performed in the Trendelenburg position with chest compression (see supplemental method), and meticulous care was taken to avoid applying negative and/or positive pressure to the inside of the lungs.

We assessed TAL efficacy in an animal model; thus, the results may not be generalisable to humans. However, since the TAL treatment proved valuable in physical removal of causative substances, it is likely that effectiveness is transferable between different animal species. In the future, we will evaluate larger animals such as pigs and sheep, and ultimately proceed to human trials. When considering clinical applications, there is no major obstruction to scale-up of the system. As for the liquid materials, PBS is widely used in clinical field. Therefore, the oxygen supply during the TAL treatment will be the primary consideration because the treatment time may be increased when using animals larger. However, rodents are the most difficult cases from the viewpoint of oxygen consumption. Indeed, oxygen consumption under anaesthesia of the rats and humans are 12–15 (mL/min/kg)^34^ and 3.21 (mL/min/kg)^35^, respectively. Furthermore, although our group is also hoping to improve the blood supply during the TAL treatment via material improvement, focusing on only the TAL treatment, we can apply TAL treatment in combination with extracorporeal membrane oxygenation (ECMO). In fact, WLL with ECMO was shown to be beneficial in that it can be performed more safely to ameliorate the respiratory failure and its causes of disease^36^. Thus, we can proceed optimistically to future prospective studies if long-term TAL treatment in rats proves successful. Another limitation was that we did not evaluate the TAL efficacy in an advanced state of respiratory failure. Moreover, respiratory failure is induced by several causes (e.g., sepsis, trauma, and pancreatitis), not only direct lung injury as in intratracheal LPS administration^3^. We will examine the values of the TAL treatment under various conditions and diseases in future research.

## Conclusions

Short-term TAL with the new system performed immediately after LPS administration successfully prevented severe pulmonary inflammation and dramatically improved survival rates. The lungs functioned normally after TAL treatment and did not require lung surfactant administration. These results suggest that short-term TAL treatment is valid for lung injuries caused by intratracheal LPS administration.

## Materials and methods

This study was approved by the local Animal Committee at the University of Yamanashi School of Medicine and conducted in compliance with the institutional animal experiment guidelines. Further details and a full description of all methods are given in the Supplemental data.

### Animal preparation

Twenty-five healthy male Sprague–Dawley rats (394 ± 28 g) were anaesthetised and intubated with a plastic tube (Surflo IV. 14 G Catheter; TERUMO CORPORATION, Tokyo, Japan). The mechanical gas ventilator and the TAL system were connected via the tube.

### Experimental design

TAL efficacy was verified in 2 lung injury models with different severities induced by changing the dose of LPS (*E. coli* O127: B8; Sigma-Aldrich Co. LLC, St. Louis, MO, USA). A severe acute lung injury model was generated using 5 mg/kg of LPS in experiment 1, and 10 mg/kg of LPS was used to create a lethal acute lung injury model in experiment 2 (Supplemental Figure S1).

In experiment 1, 15 rats were randomly divided into 3 groups: 5 rats in the TAL treatment group received 5 min of TAL and 3 h of MGV 20 min after LPS administration (LPS + TAL group); five rats in the MGV treatment group received 5 min and 3 h of MGV 20 min after LPS administration (LPS + MGV group), and 5 rats in the sham group received 5 min and 3 h of MGV 20 min after PBS administration instead of LPS (PBS + MGV group). After 2 days, the haemodynamic and blood gas parameters were measured. The lungs were removed from the chest, and BAL and histopathological analyses were performed.

In experiment 2, 10 rats were randomly divided into 2 groups of 5 rats each: the TAL treatment group (LPS + TAL group) and the MGV treatment group (LPS + MGV group). The treatments were the same as in experiment 1, and all surviving rats were sacrificed after 7 days. The rats’ lungs were subsequently isolated for histopathological analyses. The primary outcome of this experiment was the survival rate of the rats.

### Total alveolar lavage treatment

**Figure 6:**
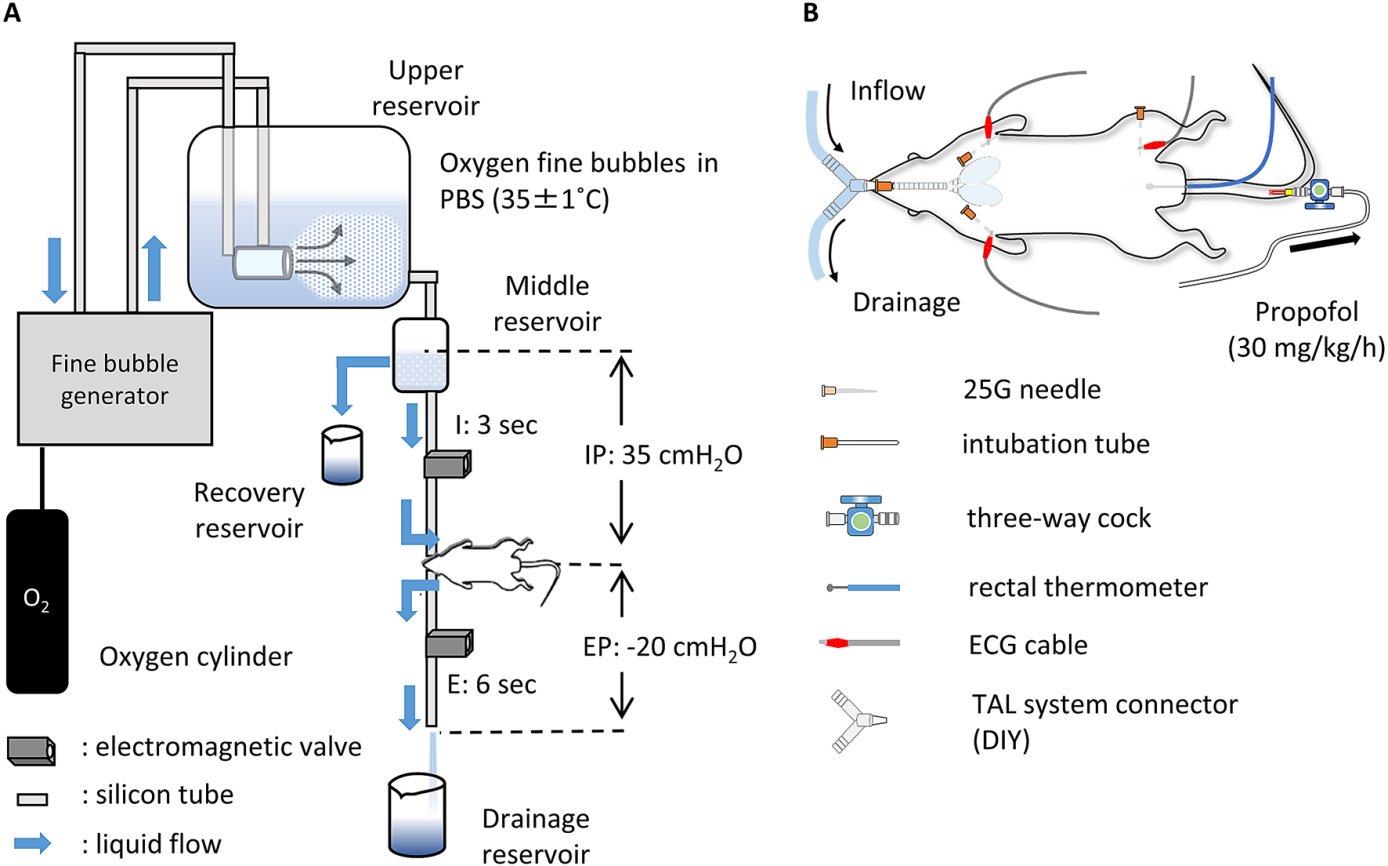
Pressure-limited time-cycled total liquid ventilation system with oxygen fine bubble dispersed saline. (**A**) Oxygen fine bubble dispersed saline (FB dispersion) was prepared in the upper reservoir with a FB generator (Ultrafine GALF FZ1N-02; IDEC Corporation, Osaka, Japan), and the FB dispersion was circulated under hydrostatic pressure. The flow speed and the hydrostatic pressure on the lungs were controlled by changing the height of the middle reservoir (inspiratory pressure [IP], 35 cmH_2_O). The respiratory pattern was controlled by adjusting the timing of opening and/or closing of the electromagnetic valves (inspiratory time [I]: expiratory time [E] = 3:6 s). Liquid drainage during the expiratory phase was performed using the siphon principle and controlled by the length of the expiratory tube (expiratory pressure [EP], − 20 cmH_2_O). (**B**) A total alveolar lavage (TAL) model rat was prepared with oral intubation under continuous anaesthesia. The Flow of FB dispersion was introduced via an intubation tube. Electrocardiography and rectal temperature measurements were performed to monitor the physiological conditions of each rat during TAL treatment.

A pressure-limited, time-cycled TAL system (shown in Fig. 6) was constructed as an improvement of a previous version^10^. In the present experiment, inspiratory and expiratory pressures were set as 35 cmH_2_O and − 20 cmH_2_O, respectively, and the respiratory ratio was set as I:E = 3:6 s. The TAL treatment was performed for 5 min. The characteristics of the FB dispersion are described in the Supplemental data.

### Statistical analysis

All results are expressed as the mean ± standard deviation. Comparisons between groups were performed with the Steel–Dwass nonparametric multiple comparison test following a Kruskal–Wallis nonparametric 1-way analysis of variance (ANOVA). Comparisons between the values at day 0 and day 2 in each group were examined with the Wilcoxon signed-rank test. The survival curves were constructed by the Kaplan–Meier method, and differences between the curves were tested with the log-rank statistic. Statistical analyses were performed using Statcel version 3 (OMS Publishing Ltd., Tokyo, Japan), and *p* values < 0.05 were considered statistically significant.

### Ethics approval and consent for participation

This study was approved by the local Animal Committee of the University of Yamanashi School of Medicine (project No. A30-9) and conducted in compliance with the institutional animal experiment guidelines. Further details and a full description of all methods are given in the Online Supplemental data.

## Supplementary information

Supplementary Information.

## Data Availability

The datasets used and/or analysed during the current study are available from the first author or corresponding author on reasonable request. Supplementary information files are also available for further information.

## Abbreviations

TAL: Total alveolar lavage
MGV: Mechanical gas ventilation
FB: Fine bubble
ARDS: Acute respiratory distress syndrome
PLV: Partial liquid ventilation
PFCs: Perfluorocarbons (liquid)
LPS: Lipopolysaccharide
PBS: Phosphate-buffered saline
BAL(F): Bronchoalveolar lavage (fluid)
PaO_2_/FIO_2_: Arterial oxygen partial pressure/fractional inspired oxygen concentration
SaO_2_: Arterial oxygen saturation
PaCO_2_: Arterial carbon dioxide partial pressure
MAP: Mean arterial pressure
PIP: Peak inspiratory pressure
ELISA: Enzyme-linked immunosorbent assay
CINC-1: Cytokine-induced neutrophil chemoattractant 1
IL-6: Interleukin-6
