# Supplementary material for: Total alveolar lavage with oxygen fine bubble dispersion directly improves lipopolysaccharide-induced acute respiratory distress syndrome of rats
Source: Sci Rep. 2020 Oct 6;10:16597. doi: 10.1038/s41598-020-73768-9 (PMC7538589; doi:10.1038/s41598-020-73768-9)
Supplement: Supplementary file 1 — Supplementary Information. [file 41598_2020_73768_MOESM1_ESM.pdf]

Supplemental data

Total alveolar lavage with oxygen fine bubble dispersion directly improves Lipopolysaccharide-induced acute respiratory distress syndrome of rats

Kenta Kakiuchi, Takehiro Miyasaka, Shinji Takeoka, Kenichi Matsuda, Norikazu Harii*

SUPPLEMENTAL METHODS

Animal preparation

Twenty-five healthy male Sprague–Dawley rats (394 ± 28 g) were anaesthetized by an intramuscular injection of 37.5 mg/kg ketamine hydrochloride (Ketalar; DAIICHI SANKYO COMPANY, LIMITED, Tokyo, Japan). Ten minutes later, 10 mg/kg of propofol (Propofol; Maruishi Pharmaceutical Co., Ltd., Osaka, Japan) was intravenously administered to induce anaesthesia further and was maintained at 30 mg/kg/h for continuous anaesthesia. Oral intubation with a 30-mm-long plastic tube (Surflo IV. 14 G Catheter; TERUMO CORPORATION, Tokyo, Japan) was performed with a flexible guidewire. The mechanical gas ventilator and total alveolar lavage (TAL) system were connected via the tube.

Respiratory management

Respiratory management was performed with an animal ventilator (SAR-830/P ventilator; CWE Inc., PA, USA) under the following conditions: fractional inspired oxygen concentration (F_IO_2), 1.0; positive end-expiratory pressure (PEEP), 3 cm H_2O; respiratory rate, 60/min; inspiratory/expiratory ratio (I:E), 1:1; and tidal volume, 9.3 ± 0.3 mL/kg. Electrocardiography and rectal temperature measurements were performed with a signal recording system (PowerLab 4/26; ADInstruments, New South Wales, Australia) for monitoring the physiological condition of the rats. The airway pressure was monitored with a

biological information monitor (BSM-2303; NIHON KOHDEN Co., Ltd., Tokyo, Japan).

Lipopolysaccharide administration

In experiment 1, 5 mg/kg (5 mg/mL in phosphate-buffered saline [PBS]) of lipopolysaccharide (LPS) was administrated into the lungs via an intubation tube using a micro sprayer (MicroSprayer, Penn-Century, PA, USA). A rat was fixed in the supine position with its head tilted at 45°, half of the dose was administered in the left lateral position, and the left chest was vibrated with an electric toothbrush (Mediclean HT-B471, OMRON, Kyoto, Japan) for 10 s. Mechanical gas ventilation (MGV) was connected for 30 s in the supine position for oxygenation. The remaining dose was administered in the right lateral position, and the right chest was vibrated with the electric toothbrush for 10 s. Finally, chest percussions and massages for both lungs were manually performed with MGV for 30 s to spread the LPS homogenously into the lungs. In experiment 2, a lethal lung injury model was prepared by administering 10 mg/kg (5 mg/mL) of LPS following the same procedures as in experiment 1.

Total alveolar lavage system

A pressure-limited, time-cycled TAL system, as represented in Figure 6, was constructed by improving the previous device[1]. In this study, inspiratory and expiratory pressures were set at 35 cm H_2O, and -20 cm H_2O, respectively, and the respiratory ratio was set at I:E = 3:6 s. These

settings were determined as the appropriate conditions to maintain a minute volume and to drain the liquid from the lungs without any lung damage due to hyperdiastolic reactions. At the beginning of the inspiratory phase, the electromagnetic valve of the inspiratory line was opened, and the expiratory line valve was closed. Oxygen fine bubble dispersed saline (FB dispersion) dispersion was introduced into the lungs through the tracheal tube for 3 s, depending on the gravity force. At the end of the inspiratory phase, conversely, the electromagnetic valve on the inspiratory line was closed, and the expiratory line valve was opened. The FB dispersion was drained from the lungs for 6 s. These procedures were repeated for 5 min. Since saline was used as the liquid material, the drained liquid did not have to be reused, unlike cases of perfluorocarbon (PFC) use[2,3], and fresh saline could be introduced for every respiration. To maintain a stable inspiratory pressure level, an overflow system was introduced in the inspiratory route. The tidal volume during TAL was calculated from body weight differences, which were automatically recorded from an electronic balance using open-source, free software (Tera Term, Japan). Since the density of the FB dispersion was nearly identical to that of normal saline (1 mg/mL), correction by density was not performed[2].

Total alveolar lavage treatment

Before starting the TAL treatment, 1.25 mg/kg of rocuronium bromide (Eslax, MSD K.K., Tokyo, Japan) was administered intravenously for myorelaxation. Then, TAL was performed

for 5 min (average tidal volume, 22.3 ± 0.65 mL/kg). After 5 min, water aspiration was performed though the tracheal tube by repeating chest compressions in the Trendelenburg position. A mechanical gas ventilator was immediately connected to the tracheal tube, and gas ventilation was performed for 3 h. Next, ventilator weaning and extubation were performed after confirming that the rat had awakened from the anaesthesia. Finally, the rats were returned to their cages with free access to water and food.

Haemodynamic and blood gas analyses

After 2 days (experiment 1) and/or 7 days (experiment 2), the surviving rats were anaesthetized with ketamine hydrochloride (37.5 mg/kg), and anaesthesia was sustained with propofol (10 mg/kg and 30 mg/kg/h) with the same protocol as described in the 'Animal preparation' section. A Tracheotomy was performed and a 14 gauge (G) × 15 mm plastic tube (Surflo IV. 14 G Catheter; TERUMO CORPORATION, Tokyo, Japan), which is shorter than the oral intubation tube, was inserted into the trachea. The animal ventilator was connected to the tracheal tube. A 24 G angiocatheter (Surflo IV. 24 G Catheter; TERUMO, Tokyo, Japan) was inserted into the carotid artery for haemodynamic monitoring and blood sampling. Haemodynamic parameters were measured with the signal recording system for 10 min. Subsequently, blood sampling (0.1 mL) was conducted from the carotid artery line, and blood gas analyses were performed with a blood gas analyser (i-STAT; Abbott, Illinois, USA). Mean

values of mean arterial pressure (MAP) and heart rate for 10 min were used as hemodynamic parameters.

Bronchoalveolar lavage

The rats were sacrificed under propofol anaesthesia after blood gas analyses. The lungs were removed from the chest, and the right main bronchus was clamped. A bronchoalveolar lavage (BAL) was performed with 5 mL of sterilized PBS for the left lung lobe through the tracheal tube. This procedure was repeated twice so that the lavage was carried out with 10 mL of PBS in total. The corrected BAL-fluid (BALF) was centrifuged (400 × *g*, 10 min, 4°C), and the cell-free supernatant was stored at -70°C for the enzyme-linked immunosorbent assay (ELISA kit; &D Systems, Minnesota, USA). We measured the amounts of interleukin-6 (IL-6; Rat IL-6 Quantikine ELISA Kit) and cytokine-induced neutrophil chemo attractant 1 (CINC-1; Rat CXCL1/CINC-1 Quantikine ELISA Kit).

Histopathological examination

After BAL examination, the right lungs were isolated and fixed by instilling 10% formalin neutral buffered solution at lower than 25 cm H_2O for 24–48 hours. The posterior and accessory lobes of the fixed lungs were embedded with paraffin and sectioned. Hematoxylin and eosin staining was performed according to the regular staining method, and the tissue

sections were observed with a digital microscope (magnification × 400) (IX71; OLYMPUS Corporation, Tokyo, Japan)[4].

Characteristics of the fine bubble dispersion

Among the fundamental properties of the FB dispersion, oxygen content, zeta potential, and the size distribution were evaluated. Oxygen FB dispersions were prepared in 5 L of PBS (DPBS; FUJIFILM Wako Pure Chemical Corp., Osaka, Japan), 5 L of saline (150 mM NaCl, prepared in-house), and 5 L of deionized water (water) using an FB generator (Ultrafine GALF FZ1N-02; IDEC Corporation, Osaka, Japan) under the following conditions: F_IO_2, 1.0; oxygen flow, 0.5 L/min; liquid temperature, 34 ± 1°C; and dissolution pressure, 270-320 kPa. Oxygen content was measured with a Clark-type oxygen electrode device (OXYG1-PLUS; Hansatech Instruments Ltd, Norfolk, UK). Zeta potential and size distribution were measured using a particle analyser (Zetasizer Nano ZS90; Malvern Panalytical Ltd, Malvern, UK).

The oxygen content of the FB dispersion in water, saline, and PBS was 40.9 mg/L, 41.7 mg/L, and 41.4 mg/L, respectively, and there were no significant differences between the oxygen levels in the different types of liquids (Supplemental Figure S2A). Considering the relationship between oxygen content in FB dispersions and the lifespan of rats outlined in the previous study[1], these values indicate a sufficient amount of oxygen to perform a 5-min TAL treatment. Supplemental Figure S2B shows the surface potentials of each FB dispersion, with

all FB samples showing a negative charge. The zeta potential in the water-FB dispersion was significantly lower (< -30 mV) than those in the other 2 samples ($p < 0.01$).

The zeta potential contributes to the stability of micro/nano particles in liquids. Electrostatic repulsion is reported to occur from ±10 mV, and the stability increases as the absolute value increases[5]. Therefore, we think that electrostatic repulsion by the surface potential did not occur in the saline-FB dispersion, whose zeta potential remained within 0 to −10 mV. The size distributions are shown in Supplemental Figure S2C to S2E; the quality of the results is slightly poor due to the low density of FBs. All results showed broad peaks and were polydispersed. Ultrafine bubbles generated from the machine were considered to have coalesced and became bigger during circulation in the container in which they were generated. Saline-FB showed the bigger size and broader distribution than the other FB dispersions, likely because its electrostatic repulsion was weaker than those of the other samples.

We used the PBS-FB dispersion for animal experiments because the PBS-FB dispersion was relatively more stable than the saline-FB dispersion and because the phosphate buffer prevented arterial blood pH reductions in the rats during the TAL treatment.

Statistical analysis for supplemental data

The results in Supplemental Figure S2 was expressed as the mean ± standard deviation. Comparisons between 3 groups were examined with the Tukey-Kramer multiple comparison

test following 1-way analysis of variance (ANOVA). The statistical analyses were performed using Statcel version 3 (OMS Publishing Ltd., Tokyo, Japan), and p values < 0.05 were considered statistically significant.

REFERENCES

1. Kakiuchi, K. *et al.* Establishment of a total liquid ventilation system using saline-based oxygen micro/nano-bubble dispersions in rats. *J. Artif. Organs* **18**, 220–227 (2015).
2. Matsuda, K., Sawada, S., Bartlett, R. H. & Hirschl, R. B. Effect of ventilatory variables on gas exchange and hemodynamics during total liquid ventilation in a rat model. *Crit. Care Med.* **31**, 2034–2040 (2003).
3. Zhu, Y.-B. *et al.* Total liquid ventilation reduces oleic acid-induced lung injury in piglets. *Chin. Med. J. (Engl).* **126**, 4282–4288 (2013).
4. Limjunyawong, N., Mock, J. & Mitzner, W. Installation and Fixation Methods Useful in Mouse Lung Cancer Research. **i**, 1–9 (2015).
5. Pate, K. & Safier, P. Chemical metrology methods for CMP quality. in *Advances in Chemical Mechanical Planarization (CMP)* (ed. Suryadevara Babu) 299–325 (Woodhead Publishing, 2016).

SUPPLEMENTAL FIGURES

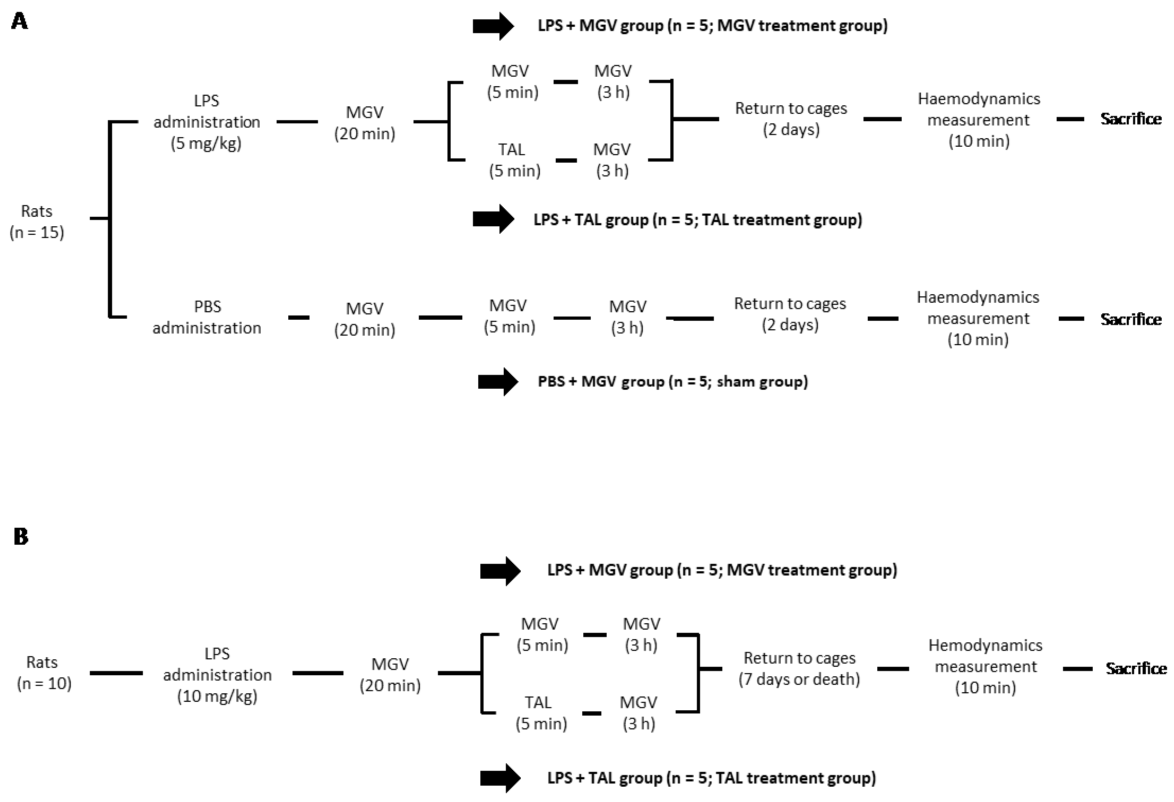

Figure S1: Summary of the experimental protocol

(A) Summary of the experimental protocol for experiment 1 is shown here. Ten rats were intratracheally administered with 5 mg/kg LPS to generate a severe lung injury model and divided into a TAL treatment group (n = 5) and an MGV treatment group as a control group (n = 5). Five rats received PBS injection instead of LPS, and served as the sham group (n = 5). Observation periods after each treatment lasted 2 days. (B) A summary of the experimental protocol for experiment 2 is shown here. Ten rats were intratracheally administered with 10 mg/kg LPS to generate a lethal lung injury model and divided into TAL treatment (n = 5) and MGV treatment groups (n = 5). Observation periods after each treatment lasted 7 days.

LPS, lipopolysaccharide; MGV, mechanical gas ventilation; PBS, phosphate buffered saline, TAL, total alveolar lavage

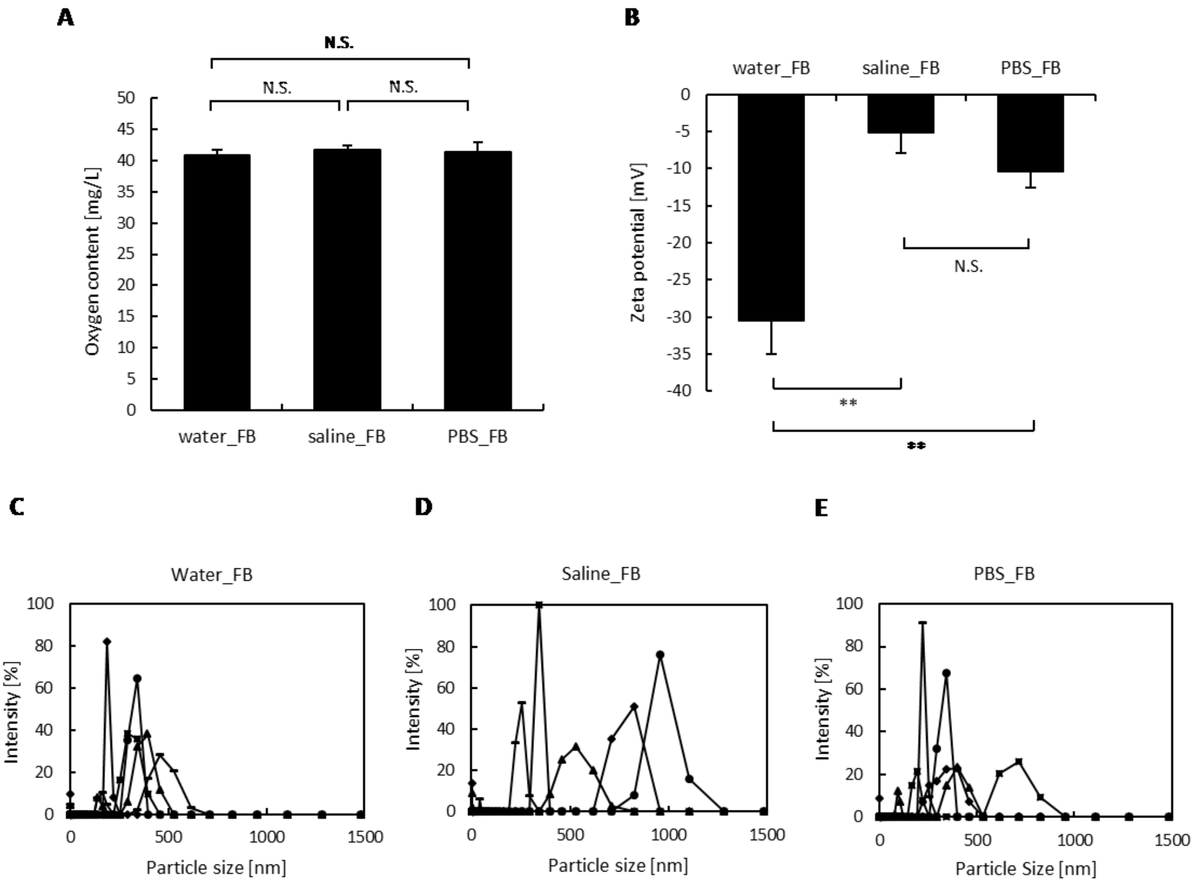

Figure S2: Fundamental properties of the oxygen fine bubble dispersed saline (FB dispersion)

(A) Oxygen content in water-FB dispersion, saline-FB dispersion, and PBS-FB dispersion samples are shown; n = 5. Data are presented as the mean ± standard deviation (SD). (B) The zeta potential of water-FB, saline-FB, and PBS-FB samples are shown; n = 4. Data are presented mean ± SD; **$p < 0.01$. The size distribution of (C) water-FB, (D) saline-FB, and (E) PBS-FB samples are shown (circle, 1st measurement; triangle, 2nd measurement; bar, 3rd measurement; diamond, 4th measurement; square, 5th measurement; n = 5).

PBS, phosphate buffered saline; N.S., not significant
